# Dietary Antioxidants and Parkinson’s Disease

**DOI:** 10.3390/antiox9070570

**Published:** 2020-07-01

**Authors:** Han-A Park, Amy C. Ellis

**Affiliations:** Department of Human Nutrition and Hospitality Management, College of Human Environmental Sciences, The University of Alabama, Tuscaloosa, AL 35487, USA; aellis@ches.ua.edu

**Keywords:** antioxidant, mitochondria, neurodegeneration, nutrient, apoptosis

## Abstract

Parkinson’s disease (PD) is a neurodegenerative disorder caused by the depletion of dopaminergic neurons in the basal ganglia, the movement center of the brain. Approximately 60,000 people are diagnosed with PD in the United States each year. Although the direct cause of PD can vary, accumulation of oxidative stress-induced neuronal damage due to increased production of reactive oxygen species (ROS) or impaired intracellular antioxidant defenses invariably occurs at the cellular levels. Pharmaceuticals such as dopaminergic prodrugs and agonists can alleviate some of the symptoms of PD. Currently, however, there is no treatment to halt the progression of PD pathology. Due to the nature of PD, a long and progressive neurodegenerative process, strategies to prevent or delay PD pathology may be well suited to lifestyle changes like dietary modification with antioxidant-rich foods to improve intracellular redox homeostasis. In this review, we discuss cellular and genetic factors that increase oxidative stress in PD. We also discuss neuroprotective roles of dietary antioxidants including vitamin C, vitamin E, carotenoids, selenium, and polyphenols along with their potential mechanisms to alleviate PD pathology.

## 1. Introduction

**Figure 1:**
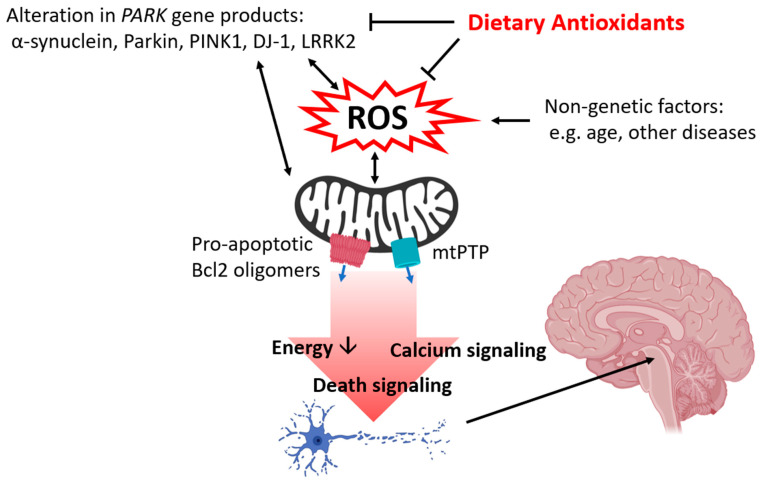
Summary of the protective roles of dietary antioxidants in Parkinson’s disease (PD). Both genetic and nongenetic factors contribute to the accumulation of oxidative stress by enhancing ROS production and impairing cellular antioxidant defense systems. Oxidative stress damages intracellular organelles, most notably the mitochondria, impairing neuronal energy metabolism and thus hindering the energy-demanding process in the brain including neurotransmission and neuritogenesis. Mitochondrial dysfunction primes apoptosis, calcium release, and opening of mtPTP which leads to the death of neurons, including the specific dopaminergic population of the SNpc which produces the signs and symptoms of PD. Illustration by BioRender.

Parkinson’s disease (PD) is a neurodegenerative disorder characterized by inadequate levels of dopamine that is caused by loss of dopaminergic neurons in the substantia nigra pars compacta (SNpc) of the basal ganglia. Dopamine also acts in other regions of the brain like the striatum, a substructure of the forebrain that regulates the motor system. Patients with PD exhibit motor symptoms including tremor, bradykinesia, rigidity, and speech difficulties, and also frequently suffer from nonmotor symptoms including depression and insomnia [1,2]. The incidence of sporadic PD is influenced by many factors including lifestyle, environment, age, and pre-existing conditions. Oxidative stress generated by many of these factors has been addressed as a major contributor to the development and progression of neurodegeneration at the cellular levels (Figure 1) [3,4,5]. In particular, mitochondrial dysfunction is a key finding in reactive oxygen species (ROS)-induced PD pathology [4,5,6,7]. Complex I, also known as NADH oxidoreductase of the electron transport chain (ETC) transfers electrons from NADH to ubiquinone and so plays a key role in oxidative phosphorylation. Complex I is vulnerable to oxidative damage, and its inhibition is also strongly associated with the generation of ROS such as superoxide and hydrogen peroxide presenting a positive feedback loop [8,9,10]. Currently, neurotoxins that target complex I like 1-methyl-4-phenyl-1,2,3,6-tetrahydropyridine (MPTP) and rotenone are used to induce parkinsonism in both in vitro and in vivo models for research, and treatment with these drugs is known to induce oxidative stress [11,12]. In addition, imbalances in dopamine metabolism contribute to ROS generation, thus damaging dopaminergic neurons. Under normal physiological conditions, dopamine is synthesized from the amino acids tyrosine and tyramine. Hydroxylation and decarboxylation of tyrosine produce dopamine, and dopamine is further converted to norepinephrine and epinephrine or undergoes degradation. However, dopamine can also undergo metabolism by monoamine oxidase (MAO) producing the highly reactive metabolite 3,4-dihydroxyphenylacetaldehyde (DOPAL) [13], and dopamine itself can undergo oxidation. Accumulation of DOPAL and oxidized dopamine increases the production of ROS damaging mitochondria [14,15,16,17,18].

## 2. Oxidative Stress and *PARK* Genes

Approximately 5-10% of PD cases are associated with abnormalities of *PARK* genes [19,20,21]. The mutation of *PARK* genes increases oxidative stress in neurons by enhancing the production of ROS or impairing intracellular antioxidant defense systems. ROS predisposes *PARK* genes to abnormal protein production and vice versa (Figure 1). α-synuclein is a protein encoded by the *SNCA (PARK1)* gene. Although α-synuclein supports synaptogenesis and synaptic plasticity during normal physiology [22,23], α-synuclein aggregation-associated neuronal damage is a common finding in PD affected tissue. Application of oligomeric α-synuclein increases ROS production and lipid peroxidation [24,25]. α-synuclein is translocated to mitochondria and directly interacts with mitochondrial proteins including ATP synthase to lower mitochondrial inner membrane potential, thus altering neuronal energy metabolism and opening mitochondrial death channels [24,26]. Increased oxidative stress induced by treatment with hydrogen peroxide or depletion of antioxidant enzymes enhances post-translational modification and aggregation of α-synuclein and worsens the progression of PD [27,28,29]. 

Parkin, the ubiquitin E3 ligase encoded by the *PARK2* gene, regulates mitochondrial quality control. Mutations of *PARK2* are the most common cause of early-onset PD. Approximately 77% of early-onset familial PD in patients younger than 30 years old have Parkin mutations [30]. Parkin works in conjunction with the myocytes lacking PTEN-induced kinase 1 (PINK1), a key enzyme responsible for carrying out autophagy, encoded by the *PARK6* gene. PINK1-mediated phosphorylation of ubiquitin activates Parkin, and this enhances the removal of unwanted mitochondria [31]. Additionally, Parkin-mediated ubiquitination also targets mitofusin and miro, key components of mitochondrial fusion and mitochondrial transport, respectively [32,33]. The deletion of Parkin or PINK1 impairs mitophagy, and failure to remove dysfunctional mitochondria increases ROS production [34]. *PARK2* knockout transgenic mice treated with chronic ethanol, a stimulator of ROS, show abnormally high superoxide accumulation and glutathione depletion [35]. Application of the mitophagy inducer, autophagy and beclin 1 regulator 1 (AMBRA1), restores mitophagy in PINK1 knockout mice and suppresses ROS production [36]. Overall, the Parkin/PINK1 system plays a critical role in regulating intracellular ROS by mitochondrial quality control, removing inefficient and damaged mitochondria. 

Protein deglycase DJ-1 is encoded by the *PARK 7* gene. Although the function of DJ-1 is less studied than other *PARK* gene products, it is reported to play an important role in supporting mitochondrial function. DJ-1 binds directly to F_1_Fo ATP synthase and the antiapoptotic protein Bcl-xL, and this interaction promotes mitochondrial energy metabolism and survival of dopaminergic neurons [37]. The depletion of DJ-1 increases the vulnerability of mitochondria to neurotoxic insults which mimic PD pathology [38,39], whereas overexpression of DJ-1 improves intracellular antioxidants and protects neurons [38,40,41]. DJ-1 plays an important role in sensing intracellular redox status during oxidative stress [42,43,44]. Under oxidative stress, DJ-1 undergoes post-translational oxidation at its Cys106 residue to form cysteine-sulfonic acid and cysteine-sulfinic acid [42,45,46], and oxidative stress also enhances translocation of DJ-1 to mitochondria. Thus, it is possible that oxidation of Cys106 may act as the signal for DJ-1 to prevent mitochondrial dysfunction during ROS production in PD. In addition, DJ-1 regulates the expression of antioxidant genes by promoting nuclear translocation of Nrf2, a transcription factor that binds to genes containing an antioxidant response element (ARE) [41,47].

LRRK2, also known as dardarin, is a kinase with guanosine triphosphatase (GTPase) and scaffolding domains [48]. LRRK2 is found in the mitochondrial membrane and interacts with other *PARK* gene products including Parkin, PINK1, and DJ-1 [49,50]. LRRK2 is encoded by the *LRRK2 (PARK8)* gene. Mutation of LRRK2 is associated with the gain of kinase activity, and this is common among patients with late-onset autosomal-dominant PD [51]. Mutation of the kinase domain of LRRK2, G2019S, exacerbates ROS-induced dopaminergic neuronal death, and application of truncated LRRK2 reverses ROS accumulation and prevents morphological alteration of these neurons [52]. In the same way, the depletion of LRRK2 or application of LRRK2 inhibitors decreases ROS, restores mitochondrial function, prevents mitochondrial fragmentation, and blocks increases in proapoptotic proteins including caspase 3, Bax, and apoptotic-inducing factor [53,54,55]. 

## 3. Oxidative Stress and Mitochondrial Dysfunction

Oxidative stress and mitochondrial dysfunction eventually lead to neuronal apoptosis during PD. Neurotoxic stimulation and ROS exposure increase the abundance of proapoptotic Bcl-2 protein Bax and Bak in the mitochondrial membrane (Figure 1). Oligomerization of proapoptotic proteins increases the permeability of the mitochondrial membrane causing the release of cytochrome c. Cytochrome c forms apoptosomes and activates executor caspases like caspase 3. Antiapoptotic proteins Bcl-2 and Bcl-xL are reported to block apoptosis by directly binding proapoptotic Bcl-2 proteins. Upregulation of proapoptotic proteins such as Bax and Bim as well as of other mechanisms including caspase activation and cytoplasmic release of cytochrome c have been reported in various PD models [54,56,57,58,59,60]. Transgenic mice lacking Bax are resistant to MPTP-induced neuronal death in the SNpc [56], and application of microRNA (miR) including miR216a and miR7 targeting Bax are protective against MPTP treatment in an in vitro and in vivo PD models [61,62]. Bcl-xL is an antiapoptotic protein that binds to DJ-1 and regulates energy metabolism in dopaminergic neurons [37,63]. A recent study shows that Bcl-xL undergoes post-translational cleavage during oxidative stress, and the accumulation of truncated Bcl-xL leads to mitochondrial dysfunction [64]. Approaches that inhibit proteolytic cleavage of Bcl-xL are reported to be protective against neurotoxicity. Treatment with antioxidants prevents the accumulation of truncated Bcl-xL and rescues neurons from oxidative stress [64]. SH-SY5Y cells derived from human bone marrow that overexpress PINK1 show decreased proteolytic cleavage of Bcl-xL by enhancing phosphorylation of Bcl-xL [65]. Bcl-xL Cre-lox knockout mice show decreased tyrosine hydroxylase-positive cells indicating loss of dopaminergic neurons in the SNpc [66]; thus, maintaining functional Bcl-xL may be critical in preventing PD-associated neuronal death.

The association between neuronal death and opening of mitochondrial permeability transition pore (mtPTP), a large less-selective mitochondrial inner membrane death channel, has been documented in PD models [24,67,68]. The opening of mtPTP allows the passage of ions and small molecules less than 1.5KDa and depolarizes the mitochondrial inner membrane. mtPTP also enhances calcium release [24] which can trigger apoptosis (Figure 1) [69]. Loss of the mitochondrial electrochemical gradient impairs ATP production by the F_1_Fo ATP synthase and impairs neuronal energy metabolism [70,71,72]. The F_1_Fo ATP synthase plays a key role in ATP production and mPTP formation [72,73,74]. The F_1_Fo ATP synthase interacts with *PARK* gene products DJ-1 and α-synuclein [24,37]. Interaction between DJ-1 and F_1_Fo ATP synthase enhances neuronal energy metabolism and promotes elongation and arborization of dopaminergic neurons [37]. On the other hand, oligomeric α-synuclein co-localizes with the F_1_Fo ATP synthase and causes oxidative modification of its β subunit, the key subunit that interacts with ADP and ATP [24]. This oxidative modification increases the opening of mtPTP. Similarly, treatment with α-synuclein, known to form insoluble fibrils during PD pathology, favors mtPTP opening in both in vitro and in vivo models, and application of the mPTP inhibitor cyclosporin A reverses α-synuclein-induced mitochondrial dysfunction [60,75]. The depletion of PINK1 decreases mitochondrial inner membrane potential and increases the opening of mtPTP, and this leads to mitophagy and neuronal death [76,77].

## 4. Neuroprotective Dietary Antioxidants 

Neurodegeneration at the cellular level develops years before patients exhibit clinical manifestations of PD. Therefore, finding strategies that can be applied over a lifetime seems of logical importance in fighting against PD. An increasing number of studies have addressed neuroprotective roles of nutrients and functional foods against neurodegeneration [78,79,80]. In particular, certain vitamins, minerals, and phytochemicals exhibit their antioxidant properties by directly scavenging ROS, binding to antioxidant enzymes as cofactors, and by regulating genes that control intracellular antioxidant systems (Figure 1). Advancing technologies in liquid chromatography and mass spectrometry such as LC/MS/MS and MALDI-TOF allow quantitative analysis of these nutrients and application of molecular approaches including sequencing, polymerase chain reaction, and electrophoresis to elucidate the association between *PARK* genes and dietary antioxidants. Here, we discuss dietary antioxidants that may potentially prevent or delay the progression of PD (Table 1).

### 4.1. Vitamin C

Vitamin C, also called ascorbic acid or ascorbate, is abundant in fruits and vegetables. Although most mammals are able to synthesize vitamin C endogenously, humans lack the necessary enzyme L-gulonolactone oxidase, so humans must ingest this essential nutrient in food or supplements [149]. Vitamin C acts as an antioxidant by donating electrons to neutralize the toxic effect of free radicals. Depending on available in vivo concentration, at high doses (≥500 mg/d), vitamin C has been shown to exhibit prooxidant properties [150]. In addition to its role in regulating cellular redox status, vitamin C supports the actions of hydroxylases involved in neurotransmitter synthesis including dopamine β-hydroxylase. Neural tissue including the brain contains high levels of vitamin C relative to other tissues, and neuroprotective roles of vitamin C have been discussed in various neurodegenerative disease models [151]. Treatment with divalent metal cations like copper and iron augment oligomerization of α-synuclein during challenge with DOPAL, a neurotoxic byproduct of dopamine metabolism [81], and treatment with vitamin C prevents α-synuclein oligomerization by inhibiting the oxidation of DOPAL [81] or interaction with copper [82]. A *Drosophila* model of PD shows increased oxidative stress with subsequent loss of dopaminergic neurons and locomotor deficits; treatment with vitamin C increased antioxidant enzyme activity and alleviated the PD-associated phenotype [99,127,152,153]. This model of PD is based on depletion of ubiquitin c-terminal hydrolase (UCH), an antioxidant enzyme, that thus enhances aging-associated degeneration of dopaminergic neurons and decreases dopamine content in the brain. The application of vitamin C (0.5 mM) compensates for these effects of UCH knockdown in *Drosophila* [152]. Vitamin C activates ten-eleven-translocation 1-3 (Tet1-3) enzymes and Jumonji C-domain-containing histone demethylases (Jmjds) [154]. These enzymes catalyze the formation of 5-hydroxymethylcytosine in DNA [155] and demethylation of lysine residues in histone, respectively. Tets and JmJds are required during the early stages of dopaminergic neuron differentiation, and treatment with vitamin C advances the development of neural stem cells derived from the embryonic midbrain [154]. Despite the protective roles of vitamin C found in in vitro and animal models, the efficacy of vitamin C against PD in humans is still controversial. Blood samples collected from PD patients show increased lipid peroxidation coupled with significantly lower levels of vitamin C compared to healthy controls [156], but some studies have also reported negligible effects of vitamin C on PD in human subjects [157]. Despite controversial results in human subjects, vitamin C may improve the therapeutic capacity of levodopa by enhancing its bioavailability and alleviating its toxic side effects [158,159]. 

### 4.2. Vitamin E

Vitamin E encompasses the tocopherols and tocotrienols found in plant sources including grains, legumes, vegetables, and seeds. Both tocopherols and tocotrienols have a chromanol ring and a hydrocarbon chain. Tocopherols have a saturated chain whereas tocotrienols contain double bonds. Vitamin E exhibits strong antioxidant properties by acting as a ROS scavenger, attenuating mitochondrial dysfunction, and preventing neuronal apoptosis during neurotoxic insults that mimic neurodegenerative disease [64,160]. Both tocopherol and tocotrienol bind to α-tocopherol transfer protein (TTP), a critical regulator of vitamin E movement and metabolism. α-tocopherol has an 8.5-fold higher affinity for TTP than α-tocotrienol [161], thus α-tocopherol is generally considered to have better bioavailability. However, studies are increasingly demonstrating that tocopherols and tocotrienols have varying roles in different tissues and microenvironments. For example, tocotrienols exhibit a stronger antioxidant capacity in lipid-rich biological membranes [162], thus tocotrienols may be effective in protecting lipid-rich organs like the brain [64,163]. Long-term intraperitoneal injection of α-tocopherol and the water-soluble analog Trolox improved long-term potentiation (LTP) and long-term depression (LTD) in PINK1 knockout mice [164]. Martella et al. report that chronic treatment with low concentration rotenone does not alter ATP production or viability of dopaminergic neurons in heterozygous PINK1 knockout (PINK1 ^+^/^−^) mice [165]. Despite this seemingly insignificant outcome, this treatment also completely impairs both LTP and LTD, and intraperitoneal injection of α-tocopherol (100 mg/kg) and Trolox (5 mg/kg) reverse this synaptic plasticity impairment [165]. DJ-1 mutant flies show altered redox homeostasis as evidenced by high levels of global ROS and hydrogen peroxide production and decreased activity of catalase and superoxide dismutase [99]. However, supplementation with α-tocopherol decreases global ROS levels in DJ-1 mutant flies [99]. In addition to tocopherols, tocotrienols protect neurons against oxidative stress-associated damage. Primary hippocampal neurons treated with α-tocotrienol show a decrease in total and mitochondrial ROS accumulation, and α-tocotrienol attenuates glutamate-induced post-translational cleavage of Bcl-xL to enhance the functions of antiapoptotic Bcl-xL [166,167]. In this study, α-tocotrienol was suggested to exert its effect by blocking the oligomerization of proapoptotic Bcl-2 proteins [167]. Oral administration of 100 μg/kg δ-tocotrienol prevents the loss of dopaminergic neurons in the SNpc and improves motor behavior in a mouse model of PD [128]. δ-tocotrienol binds to the estrogen receptor β and activates PI3K/Akt signaling pathways including phosphorylation of protein kinase B (PKB, Akt) and extracellular signal-regulated kinase (ERK) 1/2 [128,168]. Akt activates Nrf2 [169,170], and Nrf2-mediated upregulation of antioxidant and prosurvival genes is an important mechanism for the neuroprotective properties of many antioxidant nutrients [171,172,173]. Clinical studies with PD patients show that higher consumption of dietary vitamin E is inversely related to PD occurrence [157,164,174,175]. However, contrary reports have also been published on PD in human subjects [176,177]. Data from randomized controlled trials with vitamin E are limited. However, in a randomized double-blind placebo-controlled trial, Taghizadeh et al. reported significant improvement in clinical symptoms as assessed by the Unified Parkinson’s Disease Rating Scale (UPDRS) among PD patients who received 400 IU of vitamin E in combination with 1000 mg of omega-3 fatty acids [178]. These researchers also reported increases in circulating glutathione and total antioxidant capacity along with decreased high-sensitivity C-reactive protein with treatment compared to placebo. Although promising, further investigation into the specific roles of vitamin E subgroups will be important to clarify the efficacy of vitamin E in clinical disease. 

### 4.3. Vitamin A and Carotenoids

Vitamin A is a fat-soluble vitamin found in both animal (e.g., liver) and plant sources and can also be produced from provitamin A carotenoids. Vitamin A exists as multiple forms: retinol (alcohol), retinal (aldehyde), retinoic acid (carboxylic acid), and retinyl ester (ester form). Retinal binds to opsin and activates rhodopsin, a G-protein coupled receptor that senses light in the eye. Retinoic acid binds to nuclear receptors including retinoic acid receptor (RAR) and retinoid X receptor (RXR) and regulates transcription of genes that control growth and differentiation [179]. In addition to these roles, vitamin A exhibits neuroprotective properties against neurodegeneration. Retinoic acid promotes differentiation of GABAergic neurons expressing dopamine receptors [132,133,179], and changes in PD include inhibition of retinoic acid-mediated neuronal differentiation [180]. Oral supplementation with retinoic acid upregulates the μ-type opioid receptor (MOR1), a G-protein-coupled receptor that mediates inhibitory signaling, in the dorsal striatum and attenuates repetitive dyskinetic movements in PD mice [181].

Carotenoids include the yellow, orange, and red pigments found in fruits and vegetables like carrots, tomatoes, watermelons, and pumpkins, and are also found in algae, salmon, and shrimp. Examples of carotenoids include carotene, lycopene, lutein, and astaxanthin. Serum α-carotene, β-carotene, and lycopene levels are significantly decreased in PD patients, and decreased serum carotenoid levels are also associated with poorer motor function [174,182]. However, a meta-analysis that examined the association between PD and vitamin A and carotenoids (lutein, α-carotene, β-carotene, lycopene, β-cryptoxanthin, zeaxanthin and canthaxanthin) concluded that the evidence was insufficient to make an epidemiological association between vitamin A/carotenoids and risk of developing PD [183]. In an in vivo animal model, oral administration of lycopene (5–20 mg/kg) attenuates oxidative stress induced by intraperitoneal injection of MPTP in mice, and lycopene also inhibits apoptosis by decreasing Bax and caspases while increasing Bcl-2 [129]. Treatment with lutein prevents MPTP-induced Bax and caspase increases, and lutein also improves motor function in MPTP challenged mice [130]. Astaxanthin lowers intracellular ROS and improves superoxide dismutase and catalase activity, and treatment with astaxanthin prevents apoptotic death in MPTP challenged SH-SY5Y cells [100]. Astaxanthin attenuates MPTP-induced neuronal injury via the downregulation of α-synuclein [83]. miR-7 directly binds to the 3′ UTR of α-synuclein mRNA and decreases the translation of α-synuclein [184]. Treatment with astaxanthin prevents the loss of miR-7 to lower the toxic effects of α-synuclein in SH-SY5Y cells [83]. Although clinical trials are lacking, oral supplementation with astaxanthin prevents loss of neurons in the SNpc and tyrosine hydroxylase-positive cells in the striatum from intraperitoneally injected MPTP in mice [131].

### 4.4. Selenium

Selenium is an essential trace mineral-rich in Brazil nuts, seafood, and organ meats and is also found in water and soil. The selenium content of plants is directly related to the selenium content of the soil [185]. Enzymes that regulate intracellular redox status likes glutathione peroxidase and thioredoxin reductase are selenoproteins that require selenium at their active sites, and mutations of the selenocysteine residues impair enzyme activity [186]. Microarray investigation reveals that rotenone treatment downregulates the *SELENBP1* gene which encodes selenium binding protein 1, along with other genes that control apoptosis and mitochondrial function [187]. Neuroprotective functions of the selenium-containing quinoline derivative, 7-chloro-4-(phenylselanyl) quinoline, against the rotenone challenge highly correlates with selenium content in the brain of fruit flies [188]. Intraperitoneal delivery of selenium selenite (0.1, 0.2, and 0.3 mg/kg) increases glutathione peroxidase activity, alleviates lipid peroxidation, and improves motor function of the 6-hydroxydopamine challenged striatum in rats [101]. Interestingly, selenium treatment also shows dose-dependent protection of other antioxidant enzymes including glutathione reductase, glutathione transferase, and catalase [101]. Intraperitoneal injection of selenium partially improves dopamine metabolism during the MPTP challenge [189]. Analysis of soil samples from 4856 sites in the US demonstrates that higher selenium content inversely correlates with mortality from PD [190]. Human studies investigating selenium supplementation for PD are lacking. However, low plasma selenium concentrations are associated with decreased performance in neurological tests among older adults [191]. Conversely, increased levels of selenium in cerebrospinal fluid and plasma have been reported in PD patients [192,193]. Chronic exposure to selenium enhances oxidative stress in the brain and leads to cognitive impairment in animal models [194,195]. The underlying mechanism for these findings is unclear; however, evidence suggests that either a deficiency or excess of selenium may contribute to neurodegeneration or conversely PD pathology may impair mobilization of selenium in neurons. The Recommended Dietary Allowance for selenium is 55 mg/day, and the Institute of Medicine has established a Tolerable Upper Intake Level for selenium at 400 mg/day. Therefore, meeting the RDA without excess may be prudent [196]. 

### 4.5. Glutathione

Glutathione is a tripeptide of glycine, cysteine, and glutamate that is widely present in both plant and animal foods. In particular, avocados, asparagus, spinach, and amino acid-rich meat, fish and poultry are good sources of glutathione. Glutathione is a major intracellular antioxidant that reduces reactive oxygen species by being oxidized to glutathione disulfide. Glutathione is required by glutathione peroxidase during the conversion of hydrogen peroxide to water. The depletion of glutathione leads to oxidative stress-induced mitochondrial dysfunction and degeneration of dopaminergic neurons [125,134,197]. Interestingly, excess of glutathione also causes neuronal damage [134], and this may be due to the overproduction of glutathione disulfide, an oxidized form of glutathione responsible for mitochondrial dysfunction and neuronal death [198]. Strategies to support glutathione homeostasis by preventing loss of glutathione or facilitating clearance of glutathione disulfide protect the brain [163,198]. Treatment with glutathione’s precursor N-acetylcysteine (NAC) prevents oxidative stress and calcium overload and rescues neurons and other brain cells during PD-like stress [102,112,135]. Consistently, a protective effect of intravenous and oral delivery of NAC has been reported in PD patients [199,200,201]; NAC is naturally found in onions and garlic, and it is available in various dosages as an over-the-counter dietary supplement [202]. However, the best duration and concentration of supplementation to consistently show a therapeutic effect in humans has not been established [200,203]. Therefore, further investigation is required. Additionally, since oral glutathione is less bioavailable, finding nutrients that enhance the body’s ability to synthesize glutathione may also be of benefit.

## 5. Polyphenols 

Polyphenols are characterized by the presence of multiple phenol groups and a six-membered hydrocarbon ring structure. Based on the arrangement of phenol groups, hydrocarbon chain and additional functional groups, polyphenols are further classified into subgroups including flavonoids, isoflavonoids, curcuminoids, tannins, and stilbenoids. There are estimated to be over 8000 different polyphenols present in nature [204]. We will describe four well-investigated polyphenols—curcumin, resveratrol, catechin, and oleuropein—and their role in PD models. 

### 5.1. Curcumin

Curcumin, 1,7-bis(4-hydroxy-3-methoxyphenyl)-1,6-heptadiene-3,5-dione is a polyphenol found in turmeric. Curcumin scavenges biological radicals including superoxide anion, hydrogen peroxide, 1,1-diphenyl-2-picryl-hydrazyl free radical, 2,2′-azino-bis (3-ethylbenzthiazoline-6-sulfonic acid) radical, and *N*,*N*-dimethyl-*p*-phenylenediamine dihydrochloride radical [205]. In addition, treatment with curcumin (10 μM) decreases oxidation-associated protein modification including carbonylation and nitrotyrosine formation to rescue dopaminergic cells [106]. Curcumin effectively protects mitochondria from oxidative stress-associated damage [206]. Curcumin (2 μM) prevents loss of mitochondrial membrane potential and electron transfer system capacity in SH-SY5Y cells depleted with PINK1 [126]. Similarly, treatment with curcumin monoglucoside (0.25–5 μM) restores mitochondrial complex I and IV activity by decreasing the accumulation of hydroperoxides and increasing glutathione levels [103]. Curcumin exhibits antiapoptotic properties. Treatment with curcumin (5 μM) decreases ROS-induced calcium influx, lowering activation of caspase 3 and caspase 9 [104]. In addition, curcumin interferes with prodeath JNK signaling to prevent downstream apoptotic pathways including the release of cytochrome c and cleavage of procaspase 3 [103,138]. In vivo studies demonstrated antioxidant [105,144] and antiapoptotic [87,136] effects of curcumin to improve PD-associated neurobehavior [103,105,107,143,144,145,146]. Intraperitoneal injection of curcumin (200 mg/kg) attenuates rotenone-induced motor impairment in rats [143]. Male Wistar rat orally administered 5–20 mg/kg demethoxycurcumin, a derivative of curcumin, show concentration-dependent protection against rotenone challenge [105]. Demethoxycurcumin attenuates rotenone-induced oxidative stress and prevents loss of dopamine in the brain [105], and animals treated with demethoxycurcumin show improved motor function [105]. Dietary supplementation with 0.5% and 2% curcumin also show similar effects on MPTP-induced mouse PD models [137]. In addition to neuroprotection, curcumin may regulate cell differentiation and proliferation. C57BL mice transplanted with curcumin-activated mesenchymal stem cells have increased antiapoptotic Bcl-2, decreased proapoptotic Bax and caspases, and avoided the loss of dopaminergic neurons during MPTP challenge [136]. Curcumin prevents α-synuclein aggregation [87] and attenuates α-synuclein-induced cytotoxicity [85]. Curcumin derivative increases the nuclear translocation of transcription factor EB, a regulator of autophagy, potentially promoting degradation of α-synuclein [86].

### 5.2. Resveratrol

Resveratrol, 3,5,4′-trihydroxy-*trans*-stilbene is a nonflavonoid polyphenol with two aromatic ring structures. Resveratrol is found in grapes and berries, and it is also commonly consumed in red wine. Resveratrol promotes brain cell differentiation and proliferation during normal physiology [207], and it is well-described to attenuate oxidative stress-associated damage during the progression of PD pathology [108,109,110,111,112,114]. Intraperitoneally administered resveratrol (20 mg/kg) decreases lipid peroxidation, increases glutathione levels, and prevents deterioration of rat SNpc against 6-hydroxydopamine, an oxidant that causes degeneration of dopaminergic neurons [108]. Various research groups have shown that resveratrol effectively protects mitochondria by decreasing the accumulation of mitochondrial ROS, preventing mitochondrial inner membrane potential loss, restoring mitochondrial respiratory enzyme activity, regulating mitochondrial fission and fusion, and protecting mitochondrial DNA in *PARK2* mutation [109,110,111,113]. Wang et al. showed that resveratrol treatment (25 μM) increases phosphorylation of Akt and prevents rotenone-induced death of PC12 cells [111]. Akt upregulates genes containing cAMP response element (CRE) including Bcl-2 [208,209], and it inactivates proapoptotic Bad and proteolytic caspases [210]. Thus resveratrol-mediated Akt phosphorylation may hinder apoptotic death during PD-like challenges. In addition, resveratrol may alleviate *PARK* gene-associated PD pathology. Male C57BL/6 mice subjected to intragastric gavage of 100 mg/kg resveratrol attenuate the loss of dopaminergic neurons and have improved motor behavior during the MPTP challenge [88]. This same study also shows that resveratrol significantly increases protein levels of LC3-II, a key protein found in the membrane of autophagosomes, and thereby facilitates degradation of α-synuclein [88]. Resveratrol also increases microRNA-214 which potentially inhibits translation of α-synuclein [89]. Fibroblasts isolated from patients with *PARK2* mutations have increased production of whole-cell ROS and mitochondrial ROS, and treatment with resveratrol protects mitochondria and improves respiration and ATP production in these cells [109]. 

### 5.3. Catechin

Catechins are flavonoids containing two benzene rings and one dihydropyran heterocycle. Catechins are found in various herbs and fruits. Tea in particular is a good source of catechins. Four major catechins include (−)-epicatechin (EC), (−)-epicatechin-3-gallate (ECG), (−)-epigallocatechin (EGC), and (−)-epigallocatechin-3-gallate (EGCG) [211]. Catechins donate an electron from a phenolic hydroxyl group and to scavenge free radicals and thus exhibit direct antioxidant properties [212,213,214]. Catechins also improve intracellular redox status by preventing the loss of other antioxidants [116]. Treatment with 10 μM EGCG lowers the accumulation of ROS and prevents activation of caspases during hydrogen peroxide challenge and protects N27 dopaminergic cells from apoptotic death [119]. Koch et al. show that a longer brewing time tends to enhance antiradical activity in teas [215] indicating that catechins retain antioxidant properties after exposure to high temperature. Although further investigation is needed, orally supplemented catechins are shown to be delivered to the brain (0.5 nmol/g) in rats [216] and an in vitro blood–brain barrier system (BBB) shows that <10% of catechins are BBB permeable [217,218]. Various research groups have demonstrated that EGCG prevents neurotoxicity associated with α-synuclein [91,92,94,95]. EGCG chelates metal ions including Cu(II) and Fe(III) to inhibit fibrillation of α-synuclein [90,93]. EGCG (350μM) enhances the formation of stable oligomers (a less-toxic form) thus prevents the accumulation of pathological fibril [95] EGCG immobilizes α-synuclein and interferes with its oligomerization in biological membranes [96], thus EGCG helps to maintain membrane integrity [95,96]. EGCG suppresses fibrillation of γ-synuclein, a type of synuclein also found in Lewy bodies [91]. EGCG improves motor behavior in *Drosophila* by preventing mitochondrial dysfunction caused by abnormalities of LRRK2 and Parkin genes [148]. Chemically induced rodent PD models produced by injection with MPTP and 6-hydroxydopamine demonstrate PD-like symptoms like bradykinesia, and administration of 10-50 mg catechin (both oral and intraperitoneal injection) improves locomotor behavior in these animals [115,116,139]. Intraperitoneal injection of 10 or 30 mg/kg catechin restores glutathione levels and increases dopamine in the rat brain [116]. Oral supplementation with 25 mg EGCG reduces oxidative stress and preserves striatal dopamine in C57BL/6J mice challenged with MPTP [115]. C57BL/6J mice intraperitoneally injected with MPTP demonstrate PD-like symptoms including bradykinesia due to loss of SNpc dopaminergic neurons, and oral administration of EGCG (25 and 50 mg/kg) in these animals lowers proinflammatory cytokines, rescues dopaminergic neurons from death, and improves motor behavior [139]. In addition to catechins’ role inhibiting PD pathology, catechins may also support existing PD treatments. Orally administered EGCG (100 and 400 mg/kg) inhibits methylation of levodopa to improve bioavailability [219].

### 5.4. Oleuropein

Oleuropein contains hydroxytyrosol, elenolic acid, and glucose. It is a major phenolic compound found in olive oil. Although oleuropein is predominant, other oleuropein derivatives such as oleuropein aglycon and oleuroside are also found in olive oil [220]. Oleuropein acts as a scavenger of superoxide, nitric oxide, 2,2′-azinobis-(3-ethylbenzothiazoline-6-sulfonic acid, and 2,2-diphenyl-1-picrylhydrazyl radicals [221,222]. Various research groups have demonstrated that treatment with oleuropein and its derivatives inhibit the accumulation of ROS and prevent the progression of PD pathology [97,98,120]. Palazzi et al. demonstrated that in vitro incubation with oleuropein aglycone stabilizes α-synuclein monomers to prevent pathological aggregation [97]. Similarly, Mohammad-Beigi et al. show that olive fruit extracts containing oleuropein and oleuropein aglycone inhibit α-synuclein fibril elongation, decreasing cytotoxicity caused by α-synuclein oligomers [98]. In addition, oleuropein activates redox-sensitive transcription factors like Nrf2 to potentially improve intracellular antioxidant capacity via the upregulation of antioxidant genes [120,223]. Oleuropein protects mitochondria by mitigating mitochondrial superoxide production [121]. PC12 cells treated with 1-50 μM oleuropein retain mitochondrial membrane potential during the 6-hydroxydopamine challenge, and oleuropein also alleviates endoplasmic reticulum stress to protect PC12 cells from apoptotic death [141]. Oleuropein increases mitochondrial antiapoptotic Bcl-2 and decreases proapoptotic Bax and apoptotic-inducing factor [121,142]. Furthermore, oleuropein regulates phosphorylation of dynamin-related protein 1 (Drp1) [142] and LC3-II [121], key proteins that control mitochondrial fission and mitophagy, respectively. Thus, oleuropein potentially supports an optimal mitochondrial population in cells. Oral supplementation with olive leaf extract (75–300 mg/kg) significantly increases antioxidant enzymes including superoxide dismutase and glutathione peroxidase in the rat brain [123]. Rats fed with olive leaf extract are protected from loss of dopaminergic neuron during rotenone-induced mitochondrial damage, and showed improved neurobehavior [123]. Similarly, rats supplemented with extra virgin olive oil extract show decreased lipid peroxidation and increased antioxidant enzyme activities [124]. Oral administration of oleuropein is distributed to the brain 2h after ingestion [224], so oleuropein may be a key component in olive leaf and olive oil-mediated neuroprotection. 

## 6. Conclusions

Although increasing numbers of studies performed in vitro and using animal models demonstrate a potential role in dietary prevention of PD, the efficacy of nutritional intervention to do so in humans remains controversial. Epidemiological studies examining dietary intake of antioxidant micronutrients and the risk of developing PD have yielded equivocal results, and there is a paucity of data from randomized controlled trials among people with pre-existing PD. Dietary antioxidants exhibit multiple effects rather than targeting a single specific process. Vitamin C, vitamin E, and polyphenols directly interact with ROS and terminate oxidative chain reactions. Other minerals like selenium act as cofactors to support the activity of antioxidant enzymes. Many antioxidant nutrients are involved in signaling transduction and protect downstream targets of oxidative stress to alleviate the damage that promotes the development of PD. Nutrients also regulate genes that control the development, growth, and survival of dopaminergic neurons. Polyphenols like curcumin, resveratrol, catechin, and oleuropein inhibit the formation of Lewy bodies. In this review, we have described the complex cellular and molecular mechanisms of these dietary antioxidants as an important step in developing a therapeutic strategy against PD. Future clinical studies with data safety and monitoring are warranted to determine whether these antioxidant micronutrients may act individually or in synergy as a nonpharmacological means of prevention and treatment.

## Figures and Tables

**Table 1:** List of studies investigating the roles of antioxidant nutrients in PD models.

	Vit C	Vit E	Vit A & Car	Se	GSH & NAC	Cur	Res	Cat	Ole
α-synuclein	[81,82]		[83]		[84]	[85,86,87]	[88,89]	[90,91,92,93,94,95,96]	[97,98]
Oxidative stress and antioxidant	[99]	[99]	[100]	[101]	[102]	[87,103,104,105,106,107]	[108,109,110,111,112,113,114]	[93,115,116,117,118,119]	[97,98,120,121,122,123,124]
Electron transport chain					[125]	[103,126]	[109,110,113]		
Neuronal death and apoptotic pathway	[127]	[128]	[83,100,129,130,131,132,133]		[102,112,134,135]	[87,103,104,107,126,136,137,138]	[88,89,108,111,113]	[95,116,118,119,139,140]	[97,121,122,123,141,142]
Behavioral or motor function	[127]	[128]	[130]	[101]		[105,136,143,144,145,146]	[88,108,110,113,147]	[92,115,116,117,118,139,148]	[123]

Vitamin C (Vit C), vitamin E (Vit E), vitamin A (Vit A), carotenoids (Car), selenium (Se), glutathione (GSH), N-acetylcysteine (NAC), curcumin (Cur), resveratrol (Res), catechin (Cat), oleuropein (Ole).
